# Exploratory metabolomic profiling in migraine with PFO reveals dysregulated pathways and highlights indoleacrylic acid

**DOI:** 10.1016/j.isci.2026.115587

**Published:** 2026-04-03

**Authors:** Tiantian Luo, Qian Xi, Qian Wang, Yi Wang, Rui Huang, Xiaoqing Wang, Yi Zhou, Hui Huang, Jie Zeng, Cong Lu

**Affiliations:** 1 Department of Cardiology, Sichuan Provincial People’s Hospital, School of Medicine, University of Electronic Science and Technology of China, Chengdu 610031, China; 2 Structural Heart Disease Committee, Sichuan Association of Rehabilitation Medicine, Chengdu 610031, China; 3 Ultrasound Medicine and Computational Cardiology Key Laboratory of Sichuan Province, Chengdu 610031, China; 4 Department of Laboratory Medicine, Sichuan Provincial People’s Hospital, University of Electronic Science and Technology of China, Chengdu 610031, China; 5 WangJiang Hospital, Sichuan University, Chengdu 610031, China; 6 Key Laboratory of Ultrasound in Cardiac Electrophysiology and Biomechanics of Sichuan Province, Sichuan Provincial People’s Hospital, University of Electronic Science and Technology of China, Chengdu 610031, China; 7 Department of Neurology, Sichuan Academy of Medical Sciences and Sichuan Provincial People’s Hospital, University of Electronic Science and Technology of China, Chengdu 610031, China; 8 Department of Cardiology, Sichuan Provincial People’s Hospital, North Sichuan Medical College, Nanchong 637000, China

**Keywords:** clinical neuroscience, metabolomics

## Abstract

Patent foramen ovale (PFO) is increasingly linked to migraine, yet its systemic metabolic effects remain unclear. Using untargeted metabolomics, we analyzed arterial and venous plasma from migraine patients with PFO (PFO-M, *n* = 30) and venous plasma from healthy controls (*n* = 17). We identified 211 differentially expressed metabolites and multiple pathways involving branched-chain amino acids, neurosteroids, and tryptophan metabolism. The gut microbiota-derived metabolite indoleacrylic acid (IA) was inversely associated with migraine severity and was reduced in female PFO-M patients. In an independent cohort (*n* = 220), lower IA remained negatively associated with PFO-M (OR = 0.97, 95% CI 0.96–0.98), and the combination of IA and hs-CRP improved discriminative performance compared with either marker alone (AUC = 0.722). Together, these exploratory findings suggest systemic metabolic alterations in PFO-comorbid migraine and highlight IA as a candidate metabolic feature, supporting a potential role of gut-brain axis dysregulation.

## Highlights

• PFO-migraine shows altered metabolomic features in BCAAs, neurosteroids, and tryptophan
• Indoleacrylic acid (IA) is reduced and inversely associated with migraine severity
• IA combined with hs-CRP shows improved discrimination of PFO-migraine patients

## Graphical abstract

**Figure:**
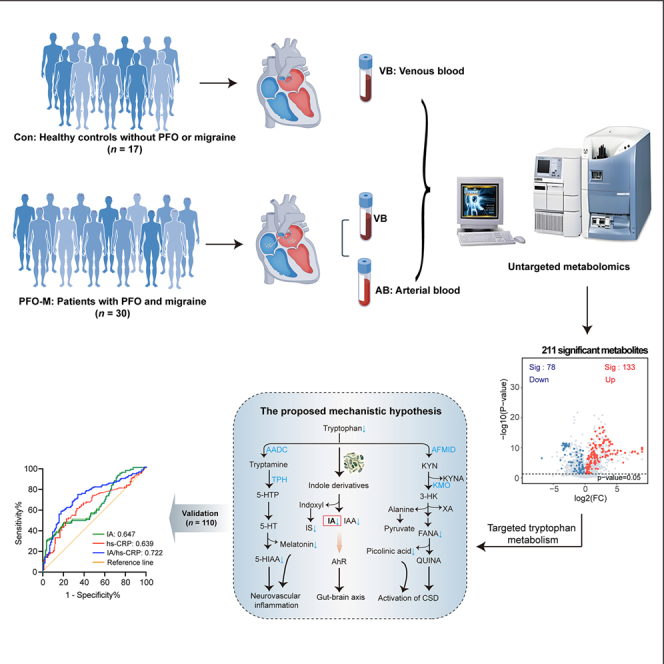

## Introduction

Migraine, an episodic brain disorder imposing a substantial societal burden,^1^ is characterized by a pathophysiological cascade involving neurogenic inflammation, cortical hyperexcitability, and trigeminovascular activation. Despite the increasing availability of therapeutic agents for migraine management, a significant proportion of patients exhibit limited treatment efficacy due to incomplete understanding of disease mechanisms, rendering them susceptible to chronic disease progression. Emerging evidence implicates patent foramen ovale (PFO), a cardiac shunt present in 15%–35% of the general population, as a potential contributor to migraine pathogenesis. Proposed mechanisms include paradoxical embolization of vasoactive substances, microemboli bypassing pulmonary filtration, and hypoxia-induced cortical spreading depression (CSD). Notably, PFO prevalence rises to 40%–60% in migraine with aura patients and correlates with enhanced headache severity and disability scores.^2^^,^^3^ As a secondary preventive measure against paradoxical embolism, percutaneous PFO closure has demonstrated reduced headache frequency and higher rates of complete migraine remission at 1-year follow-up.^4^^,^^5^^,^^6^ However, therapeutic strategies for PFO-comorbid migraine remain controversial. While PFO closure demonstrates partial efficacy in reducing migraine frequency, residual shunts persist in 26% of patients at 6 months post-procedure, with symptom recurrence predominantly observed in those with severe shunts (grade III).^7^^,^^8^ Although predictive models incorporating preoperative factors (e.g., aura status, antiplatelet use, and shunt severity) aim to optimize patient selection,^9^ the absence of mechanistic biomarkers hinders personalized intervention strategies. This gap underscores the critical need to identify metabolic signatures bridging PFO pathophysiology to migraine symptomatology.

Contemporary metabolomic studies reveal systemic metabolic dysregulation in migraine, including elevated fatty acids and lysophosphatidylethanolamine.^10^ Dysregulated tryptophan metabolism, particularly gut microbiota-derived indole derivatives, has been implicated in neurovascular inflammation and oxidative stress.^11^^,^^12^ Additionally, perturbations in branched-chain amino acids (BCAAs) and neurosteroids are linked to mitochondrial dysfunction and neurotransmitter imbalances (e.g., glutamate, γ-aminobutyric acid (GABA)).^13^^,^^14^^,^^15^ Despite these advances, few prior studies have characterized the systemic metabolic alterations underlying migraine pathophysiology in the context of PFO. Critical knowledge gaps persist: (1) Do these patients exhibit distinct plasma metabolic signatures compared to controls? (2) Which pathways and candidate metabolic features link metabolic perturbations to symptom severity? (3) Are sex or aura status associated with these metabolic phenotypes? (4) Could metabolomic profiling inform patient stratification in the context of PFO closure?

This study bridges these gaps by employing integrated plasma metabolomic profiling. These results suggest distinct metabolic features involving disrupted BCAA catabolism, impaired neurosteroid sulfation, and dysregulated tryptophan metabolism in PFO-M. The identified metabolic signatures, particularly IA reduction, may contribute to improved characterization of patient stratification and inform future efforts toward more individualized management strategies.

## Results

### Metabolome overview comparing PFO-M and controls

**Figure 1:**
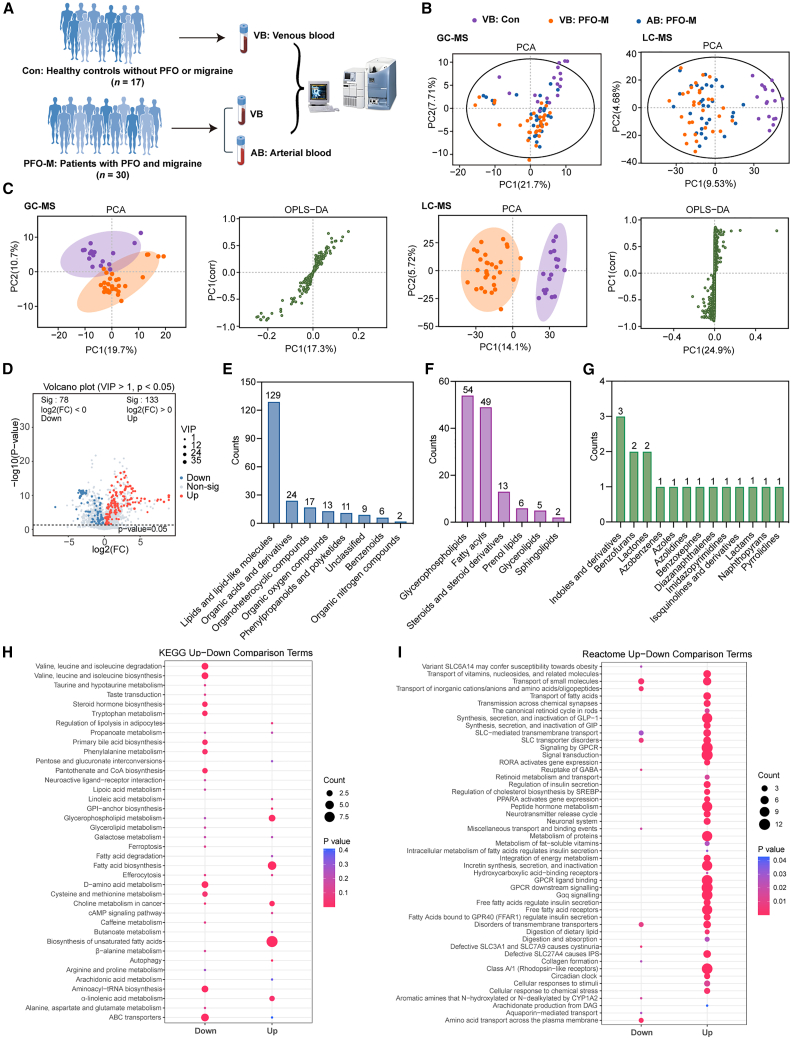
Systemic metabolomic profiling distinguishes PFO-M from healthy controls (A) Schematic of the study workflow integrating paired arterial–venous sampling, LC-MS/MS and GC-MS, differential metabolite identification, and pathway analysis. (B) Principal-component analysis (PCA) showing clustering of arterial plasma from PFO-M patients (blue), venous plasma from PFO-M patients (orange), and venous plasma from healthy controls (purple). (C) PCA and OPLS-DA score plots of venous plasma metabolites showing separation between PFO-M and controls; the S-plot highlights metabolites contributing most to group discrimination. (D) Volcano plot of differentially expressed metabolites between PFO-M and controls, showing upregulated (red, *n* = 133) and downregulated (blue, *n* = 78) metabolites. (E) Superclass classification (HMDB level 1) of the 211 differentially expressed metabolites. (F and G) Subclass distributions of lipid species and organoheterocyclic compounds, respectively. (H and I) KEGG and Reactome pathway enrichment analysis showing the significantly perturbed metabolic routes. Circle size denotes pathway impact value.

To clarify potential differences in metabolic states between the two groups, plasma metabolites were detected using LC-MS/MS and GC-MS from 30 migraine patients with PFO, termed PFO-M in this study, and 17 age- and sex-matched healthy controls (Figure 1A). The comprehensive demographic and clinical parameters of all participants are showed in Table 1. No statistical differences were observed in terms of gender, age, BMI, smoking status or comorbidities, including diabetes, hypertension or hyperlipidemia between the two groups. Notably, PFO-M exhibited elevated fasting glucose and higher cerebral infarction (CI) rates. Conversely, white blood cell, hemoglobin (HGB), platelets, and serum urea acid levels were modestly reduced but within normal ranges.

**Table 1:** Baseline demographic and clinical characteristics of participants included in the untargeted metabolomics analysis cohort

Variable	Con (*n* = 17)	PFO-M (*n* = 30)	*p* value
Gender, female, n (%)	11 (64.7%)	23 (76.7%)	0.378
Age (years)	42.24 ± 11.03	45.10 ± 8.19	0.316
BMI (kg/m^2^)	21.78 ± 2.04	22.96 ± 3.32	0.189
Smokers, n (%)	2 (11.8%)	4 (13.3%)	0.877
Diabetes, n (%)	0	1 (3.3%)	0.447
Hypertension, n (%)	0	3 (10.0%)	0.178
Hyperlipidemia, n (%)	1 (5.9%)	2 (6.7%)	0.916
Cerebral infarction, n (%)	0	9 (30.0%)	0.012
White blood cells, ×10^9^/L	6.53 ± 1.31	5.44 ± 1.28	0.008
HGB, g/L	143.41 ± 10.83	132.67 ± 15.45	0.015
Platelets, ×10^9^/L	217.41 ± 28.04	188.97 ± 59.06	0.031
Triglyceride (mmol/L)	1.41 ± 0.80	1.62 ± 1.42	0.574
Total cholesterol (mmol/L)	4.46 ± 0.32	4.28 ± 0.89	0.316
LDL-C (mmol/L)	2.62 ± 0.25	2.52 ± 0.66	0.427
HDL-C (mmol/L)	1.45 ± 0.21	1.35 ± 0.29	0.167
Glucose (mmol/L)	4.57 ± 0.34	5.17 ± 1.04	0.006
Urea acid (μmol/L)	335.94 ± 55.17	276.00 ± 51.04	<0.001
Serum creatinine (μmol/L)	61.11 ± 9.52	63.75 ± 12.74	0.462
LA (mm)	30.59 ± 3.76	30.73 ± 3.48	0.894
RA (mm)	41.59 ± 4.23	40.47 ± 3.79	0.354

Categorical data are shown as count and percentage. Quantitative data are shown as mean ± SD.

Untargeted metabolomics sequencing analysis was performed and a comprehensive dataset of 4,858 detectable metabolites was generated. PCA analysis showed a tendency to separate controls, while arterial and venous blood metabolic profiles exhibited strong intra-individual concordance (Figure 1B). OPLS-DA of venous blood metabolites suggested a clear separation trend between PFO-M patients and controls, identifying 211 differentially expressed metabolites (DEMs) (VIP >1.0, *p* value <0.05 and q < 0.10 using FDR correction; 133 upregulated, 78 downregulated, Figures 1C and 1D). Hierarchical classification of these discriminatory metabolites demonstrated lipid-associated species constituting the largest proportion (*n* = 129), followed by organic acids and derivatives (*n* = 24), organoheterocyclic compounds (*n* = 17) and others (Figure 1E). Lipid subtyping further delineated 5 major subclasses: glycerophospholipids (41.86%), fatty acyls (37.98%), steroids and steroid derivatives (10.08%), prenol lipids (4.65%), glycerolipids (3.88%), and sphingolipids (1.55%) (Figure 1F). Notably, targeted analysis of indoles-containing organoheterocyclic compounds showed that tryptophan-derived metabolites indoleacrylic acid (IA, FC = 0.60, *p* = 5.68 × 10^−10^) and indoxyl sulfate (IS, FC = 0.52, *p* = 1.2 × 10^−3^) were markedly depleted (Figure 1G).

KEGG pathway enrichment based on total differentially expressed metabolites identified six significantly dysregulated pathways after FDR correction (q < 0.1), with an additional seven pathways showing borderline significance (0.1 ≤ q < 0.2). These pathways included the valine, leucine and isoleucine degradation and biosynthesis, steroid hormone biosynthesis, tryptophan metabolism, phenylalanine metabolism, pantothenate and CoA biosynthesis, glycerophospholipid metabolism, fatty acid biosynthesis, D-amino acid metabolism, choline metabolism in cancer, biosynthesis of unsaturated fatty acids, aminoacyl-tRNA biosynthesis and ABC transporters (Figure 1H). Meanwhile, the reactome analysis implicated upregulated synaptic transmission, the canonical retinoid cycle in rods, SLC-mediated transmembrane transport, neurotransmitter release cycle, neuronal system, GPCR signaling, Gαq signaling and cellular responses to stimuli, with concomitant suppression of GABA reuptake (Figure 1I).

### PFO-M exhibited altered metabolic abundance patterns

**Figure 2:**
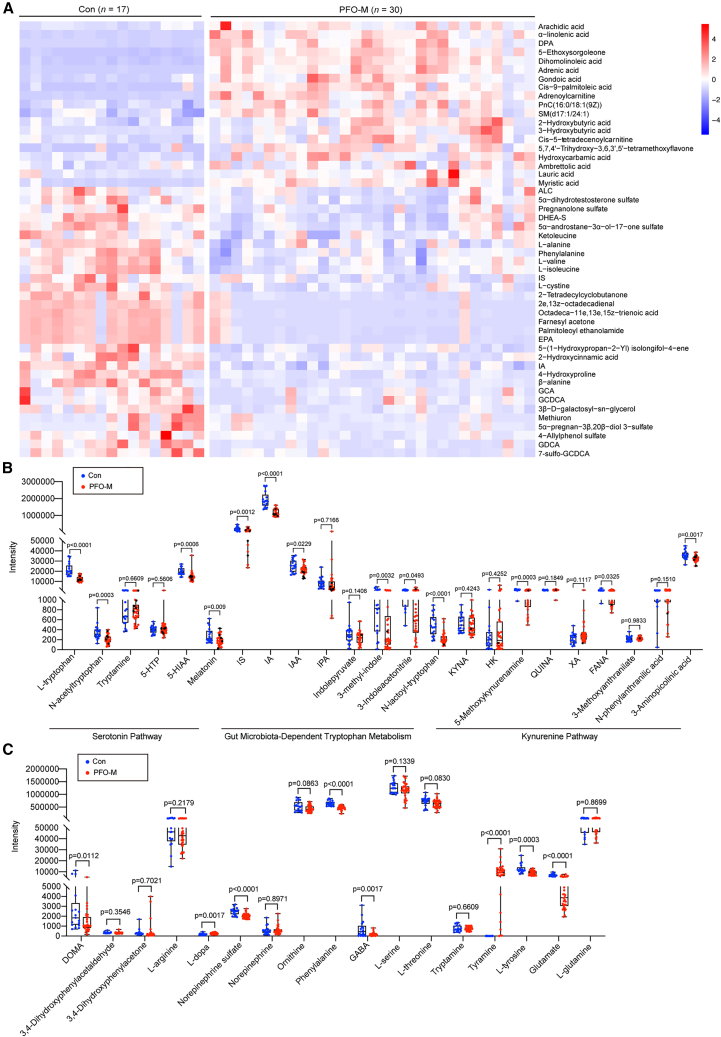
Metabolomic profiling reveals altered tryptophan metabolites and neurotransmitters in PFO-M (A) Hierarchical clustering heatmap of 50 selected metabolites related to neuroinflammatory and metabolic pathways in PFO-M (*n* = 30) and healthy controls (*n* = 17). Colors represent *Z* score-normalized metabolite abundances (blue, low; red, high). (B) Relative abundances of tryptophan-derived metabolites in PFO-M and controls. (C) Relative abundances of neurotransmitter-related metabolites. Data are shown as mean ± SEM. Significance is determined by Student’s *t* test.

To identify the most influential metabolites, we applied stringent criteria (VIP >1.0, *p* value <0.05, *and q < 0.10 using FDR correction*), yielding 211 dysregulated metabolites. From these, 50 metabolites exhibiting high abundance and putative biological relevance to neuroinflammatory pathways were selected for hierarchical clustering analysis and plotted as a heatmap. As shown in Figure 2A, 9 amino acids and derivatives, including acetyl-L-carnitine (ALC, −39.79%), ketoleucine (−20.32%), L-alanine (−25.48%), phenylalanine (−28.80%), L-valine (−22.82%), L-isoleucine (−34.51%), L-cystine (−56.61%), 4-hydroxyproline (−60.37%) and β-alanine (−69.48%), 2 indoles and derivatives, including IA (−35.74%) and IS (−47.66%), 5 steroids, including 5α-dihydrotestosterone sulfate, pregnanolone sulfate (−43.31%), dehydroepiandrosterone sulfate (DHEA-S, −46.24%), 5α-androstane-3α-ol-17-one sulfate (−43.39%) and 5α-pregnan-3β,20β-diol 3-sulfate (−71.02%), and 4 bile acids, including glycocholic acid (GCA, −60.39%), glycochenodeoxycholic acid (GCDCA, −54.35%), deoxycholic acid conjugated with glycine (GDCA, −79.00%) and glycochenodeoxycholic acid 7-sulfate (7-sulfo-GCDCA, −76.42%), were significantly lower in the plasma of PFO-M. Conversely, levels of ketogenesis markers, including 2-hydroxybutyric acid, 3-hydroxybutyric acid and *cis*-5-tetradecenoylcarnitine, were significantly increased (+68.68%, +307.60%, and +112.15%, respectively), as compared to controls.

While differential metabolites were stringently defined based on FDR-adjusted significance, we additionally performed an exploratory, hypothesis-generating analysis to examine broader metabolic patterns using nominal criteria (VIP >1 and *p* < 0.05, uncorrected). In this exploratory analysis, dysregulated tryptophan metabolism and neurotransmitter imbalances emerged as key features of migraine biology in the context of PFO. Notably, PFO-M exhibited significantly reduced levels of 23 tryptophan metabolites, including L-tryptophan, N-acetyltryptophan, 5-hydroxyindoleacetic acid (5-HIAA), melatonin, IS, IA, indoleacetic acid (IAA), 3-methyl-indole, 3-indoleacetonitrile, N-lactoyl-tryptophan, 5-methoxykynurenamine, formylanthranilic acid (FANA), and 3-aminopicolinic acid (Figure 2B). Concurrently, 17 neurotransmitters, including 3,4-dihydroxymandelic acid (DOMA), L-dopa, norepinephrine sulfate, phenylalanine, GABA, tyramine, L-tyrosine, and glutamate were differentially regulated (Figure 2C). These findings are consistent with pathway enrichment results, suggesting a potential role of tryptophan metabolism (e.g., IA^16^ and 5-HIAA^17^) and neurotransmitter pathways (e.g., GABA^18^) in PFO-M, while highlighting their possible context-dependent modulation across disease phases or subgroups.

### Correlation between differential metabolites and clinical migraine symptoms

**Figure 3:**
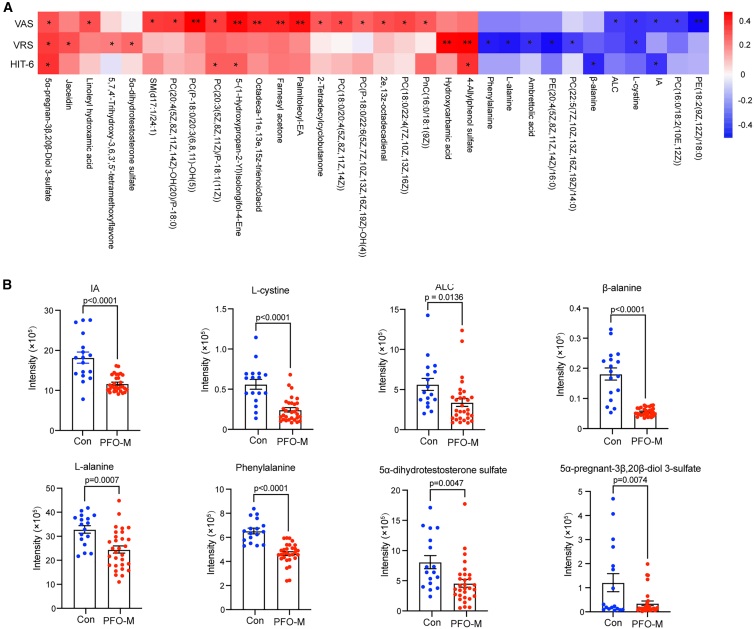
Venous plasma metabolites correlate with migraine severity in PFO-M (A) Heatmap of Spearman correlations between venous plasma differential metabolites and migraine severity scores (VAS, VRS, and HIT-6) in PFO-M patients (*n* = 30) (without multiple-testing correction). Color scale represents correlation strength (0–0.4). (B) Relative venous plasma levels of IA, ALC, L-cystine, β-alanine, L-alanine, phenylalanine, 5α-dihydrotestosterone sulfate, and 5α-pregnan-3β,20β-diol 3-sulfate. Data are shown as mean ± SEM. ∗*p* < 0.05, ∗∗*p* < 0.01 by Spearman correlation.

Spearman correlation analysis of 211 DEMs identified 32 venous plasma metabolites showing nominally significant associations with migraine severity metrics (VAS, VRS, or HIT-6) within the PFO-M cohort. Metabolites were considered associated with migraine severity if a statistically significant correlation (*p* < 0.05, uncorrected) was observed with at least one of the three severity measures (Table S1 and Figure 3A). Functional annotation of these candidate metabolites highlighted 8 metabolites with known biological roles in pathways implicated in migraine pathophysiology. As displayed in Figures 3A and 3B, PFO-M exhibited significantly reduced plasma levels of IA, a tryptophan-derived metabolite with anti-inflammatory and antiplatelet properties,^16^^,^^19^ alongside reduced ALC, L-cystine, β-alanine, L-alanine, and phenylalanine, which are considered as the modulators in neuroprotection, antioxidation, and energy metabolism,^20^^,^^21^ with these reductions being inversely correlated with symptom severity scores. Conversely, residual levels of sulfated neurosteroids 5α-dihydrotestosterone sulfate and 5α-pregnan-3β,20β-diol 3-sulfate showed positive correlations with symptom severity, potentially reflecting compensatory neuroendocrine adaptations during chronic pain progression.^22^

### Correlation analysis between arterial blood metabolites and migraine symptoms

**Figure 4:**
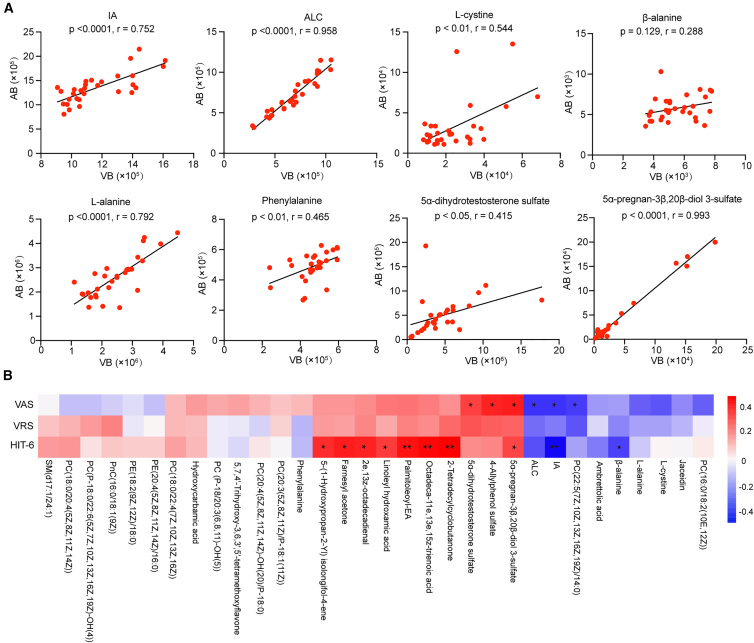
Arterial-venous concordance and arterial metabolite correlations with migraine (A) Spearman correlations between paired arterial and venous plasma metabolite levels in PFO-M patients (*n* = 30). Correlation coefficients (r) and *p* values are shown. (B) Heatmap of Spearman correlations between arterial plasma metabolites and migraine severity scores (VAS, VRS, and HIT-6) (without multiple-testing correction). ∗*p* < 0.05, ∗∗*p* < 0.01 by Spearman correlation.

Paired arterial-venous metabolomic profiling in PFO-M revealed a high degree of arterial-venous concordance (Figure 4A), supporting the validity of venous sampling for central nervous system (CNS) metabolic studies. Notably, focusing on metabolites showing venous-migraine symptoms associations, we analyzed their concentrations in arterial blood counterparts against migraine severity (Figure 4B). Consistent with the venous findings, β-alanine, IA and ALC showed inverse associations with VAS and HIT-6 scores. Additionally, 5α-dihydrotestosterone sulfate and 5α-pregnan-3β,20β-diol 3-sulfate showed positive associations with migraine intensity and disability scores, which may reflect their potential relevance to blood-brain barrier (BBB)-related processes and neuromodulatory regulation.

### Gender stratification reveals differential metabolic pathways in PFO-M

Subgroup analysis of 30 PFO-M stratified by sex revealed a female predominance (76.67% vs. 23.33%, *p* < 0.001), consistent with epidemiological trends.^23^ Baseline characteristics showed no sex differences in age, BMI, comorbidities (hypertension, diabetes, hyperlipidemia, and CI), hematological parameters (cholesterol, uric acid), PFO anatomy (tunnel length or width, RLS severity), or migraine phenotypes (aura prevalence, onset age, duration, and disability scores) (Table 2). Males exhibited a higher proportion of smokers compared to females, which aligns with global gender-specific behavioral patterns. Additionally, the elevated levels of HGB and creatinine in males may reflect physiological sex differences in erythropoiesis and muscle mass, whereas the higher triglycerides and fasting glucose in males due to the reduced insulin sensitivity and increased lipolysis driven by androgen.

**Table 2:** Baseline characteristics of PFO-M stratified by gender

Variable	Male (*n* = 7)	Female (*n* = 23)	*p* value
Age (years)	46.14 ± 8.30	44.78 ± 8.32	0.708
BMI (kg/m^2^)	22.45 ± 3.12	23.12 ± 3.42	0.646
Smokers, n (%)	3 (42.86%)	1 (4.35%)	0.009
Diabetes, n (%)	0 (0%)	1 (4.35%)	0.575
Hypertension, n (%)	1 (14.29%)	2 (8.7%)	0.666
Hyperlipidemia, n (%)	0 (0%)	2 (8.7%)	0.419
Cerebral infarction, n (%)	2 (28.57%)	7 (30.43%)	0.925
White blood cells, ×10^9^/L	5.82 ± 1.55	5.32 ± 1.21	0.372
Hemoglobin, g/L	154.29 ± 7.99	126.09 ± 10.16	<0.001
Platelets, ×10^9^/L	204.29 ± 62.42	184.30 ± 58.63	0.443
Triglyceride (mmol/L)	2.61 ± 2.50	1.32 ± 0.74	0.032
Total cholesterol (mmol/L)	3.97 ± 1.42	4.37 ± 0.68	0.306
LDL-C (mmol/L)	2.59 ± 0.72	2.49 ± 0.65	0.748
HDL-C (mmol/L)	1.17 ± 0.31	1.40 ± 0.26	0.070
Glucose (mmol/L)	5.98 ± 1.43	4.92 ± 0.77	0.016
Urea acid (μmol/L)	307.43 ± 66.03	266.43 ± 42.83	0.061
Serum creatinine (μmol/L)	81.40 ± 3.63	58.37 ± 8.99	<0.001
LA (mm)	30.00 ± 3.83	30.96 ± 3.43	0.534
RA (mm)	40.29 ± 5.02	40.52 ± 3.46	0.888
ASA, n (%)	0 (0%)	1 (4.35%)	0.575
PFO width (mm)	1.56 ± 0.93	1.49 ± 0.67	0.826
PFO length (mm)	12.50 ± 5.60	8.68 ± 4.11	0.058
With aura, n (%)	1 (14.29%)	9 (39.13%)	0.222
Age at onset of migraine (years)	33.14 ± 16.35	33.70 ± 10.87	0.918
Duration prior to enrollment (years)	12.71 ± 12.31	10.22 ± 9.52	0.575
VAS scores	6.86 ± 2.34	7.48 ± 1.67	0.435
VRS scores	2.43 ± 0.53	2.43 ± 0.51	0.978
HIT-6 scores	61.00 ± 4.00	62.61 ± 6.87	0.563

Categorical data are shown as count and percentage. Quantitative data are shown as mean ± SD.

**Figure 5:**
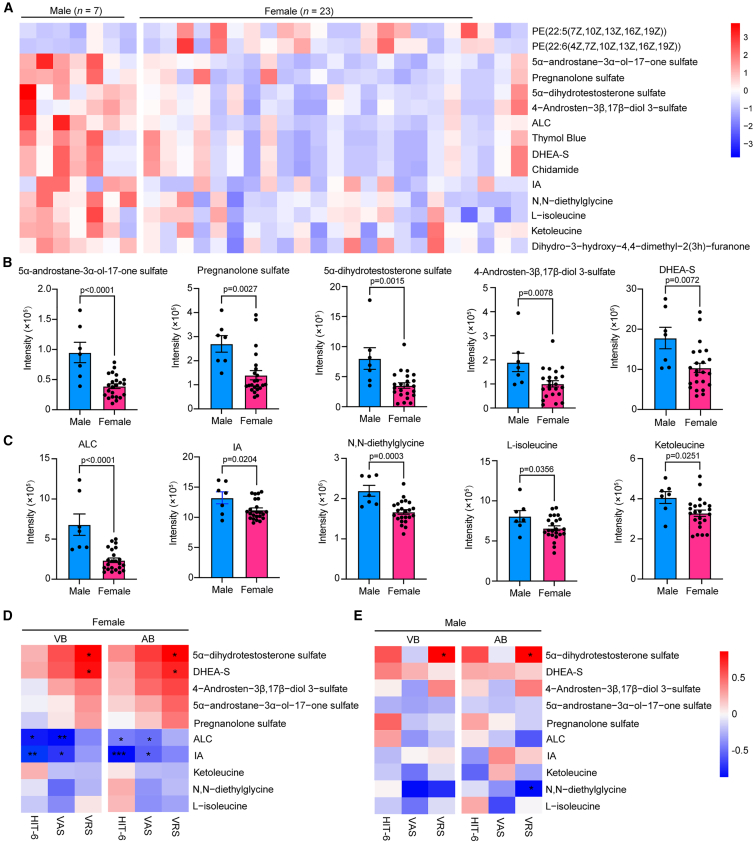
Sex-specific metabolic alterations in PFO-M (A) Heatmap of 15 sex-associated metabolites in female (*n* = 23) and male (*n* = 7) PFO-M patients. Values are *Z* score-normalized metabolite intensities. (B and C) Sex-stratified comparisons of plasma levels of 10 selected metabolites. (D and E) Spearman correlations between venous and arterial metabolite levels and migraine severity scores in females and males. Data are shown as mean ± SEM. ∗*p* < 0.05, ∗∗*p* < 0.01, ∗∗∗*p* < 0.001 by Spearman correlation.

In this female-predominant PFO-M, untargeted metabolomic profiling identified 15 metabolites showing sex-specific alterations (Figure 5A). Females exhibited markedly reduced levels of sulfated neurosteroids, including 5α-androstane-3α-ol-17-one sulfate, pregnanolone sulfate, 5α-dihydrotestosterone sulfate, 4-androsten-3β,17β-diol 3-sulfate, and DHEA-S (Figure 5B). Lower plasma levels of ALC, IA, N,N-diethylglycine, L-isoleucine, and ketoleucine were also observed in female patients compared with males (Figure 5C). We further analyzed the correlation between plasma levels of these ten metabolites with migraine severity scores. In female patients, venous and arterial plasma levels of IA and ALC showed inverse associations with HIT-6 and VAS scores, while 5α-dihydrotestosterone sulfate and DHEA-S showed positive associations with VRS scores (Figure 5D). In male patients, 5α-dihydrotestosterone sulfate levels were positively associated with VRS, whereas N,N-diethylglycine showed a negative association with the same parameters (Figure 5E). These sex-specific associations suggest potential differences in pathophysiological mechanisms, with female-specific neurosteroid-gut-brain axis dysregulation versus male-predominant glycine-androgen metabolic remodeling. Given the limited sample size in sex-stratified subgroups, particularly among male patients, these exploratory findings require confirmation in larger, sex-balanced cohorts.

### Distinct plasma metabolomic profiles in PFO-M with aura

**Figure 6:**
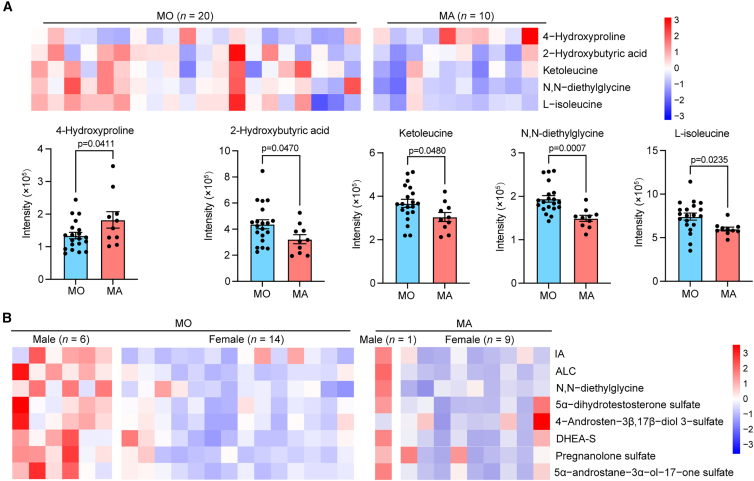
Metabolic differences associated with migraine aura in PFO-M (A) Heatmap and bar plots of five significantly altered metabolites in PFO-M without aura (MO, *n* = 20) and with aura (MA, *n* = 10). (B) Sex-stratified heatmap of eight metabolites in MO males (*n* = 6) and females (*n* = 14), and in MA males (*n* = 1) and females (*n* = 9). Data are shown as mean ± SEM.

To explore metabolic differences associated with migraine aura and to identify candidate metabolic features, the cohort of PFO-M was stratified into subgroups based on the presence (MA, *n* = 10) or absence (MO, *n* = 20) of aura symptoms. Comparative analysis of baseline clinical characteristics demonstrated no statistically significant differences between subgroups across demographic variables (age, sex, and BMI), lifestyle factors (smoking status), comorbidities, hematologic parameters, atrial dimensions, PFO morphological features, and headache severity scores (Table 3). Subsequent metabolomic profiling revealed higher plasma levels of 4-hydroxyproline and lower levels of 2-hydroxybutyric acid, ketoleucine, N,N-diethylglycine, and L-isoleucine in MA compared to MO (VIP >1.0, *p* < 0.05, *and FDR-adjusted q < 0.10*; Figure 6A). These perturbations in BCAAs catabolism and fatty acid oxidation imply a potential mechanistic role for these pathways in driving the neurovascular dysfunction underlying migraine aura pathogenesis.

**Table 3:** Baseline characteristics of PFO-M based on presence of aura

Variable	With aura (*n* = 10)	Without aura (*n* = 20)	*p* value
Age (years)	42.20 ± 8.65	46.55 ± 7.77	0.629
Gender, female, n (%)	9 (90%)	14 (70%)	0.222
BMI (kg/m^2^)	22.25 ± 3.26	23.32 ± 3.36	0.413
Smokers, n (%)	1 (10%)	3 (15%)	0.704
Diabetes, n (%)	1 (10%)	0 (0%)	0.150
Hypertension, n (%)	2 (20%)	1 (5%)	0.197
Hyperlipidemia, n (%)	0 (0%)	2 (10%)	0.301
Cerebral infarction, n (%)	3 (30%)	6 (30%)	1.000
White blood cells, ×10^9^/L	5.07 ± 0.79	5.62 ± 1.45	0.278
Hemoglobin, g/L	132.60 ± 12.26	132.70 ± 17.122	0.987
Platelets, ×10^9^/L	180.40 ± 49.45	193.25 ± 64.01	0.583
Triglyceride (mmol/L)	1.18 ± 0.86	1.84 ± 1.60	0.233
Total cholesterol (mmol/L)	4.45 ± 0.69	4.20 ± 0.98	0.486
LDL-C (mmol/L)	2.50 ± 0.62	2.53 ± 0.69	0.912
HDL-C (mmol/L)	1.47 ± 0.28	1.28 ± 0.28	0.088
Glucose (mmol/L)	4.78 ± 0.57	5.37 ± 1.17	0.149
Urea acid (μmol/L)	266.70 ± 52.38	280.65 ± 51.06	0.490
Serum creatinine (μmol/L)	58.20 ± 12.93	66.52 ± 12.93	0.092
LA (mm)	30.00 ± 3.80	31.10 ± 3.35	0.424
RA (mm)	39.50 ± 3.98	40.95 ± 3.69	0.331
ASA, n (%)	1 (10%)	0 (0%)	0.150
PFO width (mm)	1.64 ± 0.66	1.44 ± 0.75	0.472
PFO length (mm)	8.97 ± 3.82	9.87 ± 5.14	0.629
Age at onset of migraine (years)	27.80 ± 14.58	36.45 ± 9.71	0.062
Duration prior to enrollment (years)	13.90 ± 12.46	7.70 ± 2.11	0.239
VAS scores	7.70 ± 2.11	7.15 ± 1.66	0.442
VRS scores	2.6 ± 0.52	2.35 ± 0.49	0.206
HIT-6 scores	65.20 ± 5.16	60.75 ± 6.39	0.067

Categorical data are shown as count and percentage. Quantitative data are shown as mean ± SD.

To explore potential sex-related patterns within aura-defined subgroups, exploratory sex-stratified analyses were performed (Figure 6B). In the non-aura subgroup, female patients (*n* = 14) showed lower venous plasma levels of several metabolites compared with males (*n* = 6), including IA, ALC, N,N-diethylglycine, and multiple sulfated neurosteroids. Given the limited sample size, these differences should be interpreted descriptively rather than as statistically robust findings. In the aura subgroup, the small number of male patients (*n* = 1) precluded meaningful sex-stratified statistical analysis; however, exploratory inspection suggested a consistent downward trend in IA and ALC levels among female patients (*n* = 9) with aura. Collectively, these findings suggest that sex-specific metabolic differences may be associated with aura-related phenotypes in PFO-migraine patients, with reduced IA and ALC levels identified as candidate features particularly observed in females.

### Relevance of plasma IA levels to PFO-M

**Figure 7:**
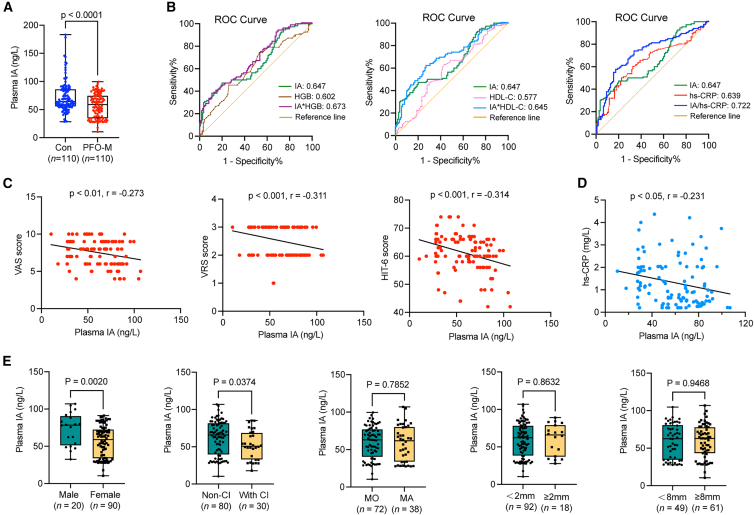
Validation and clinical relevance of IA in PFO-M (A) Plasma IA levels measured by ELISA in PFO-M (*n* = 110) and healthy controls (*n* = 110). (B) ROC curves for IA, HGB, HDL-C, hs-CRP, and their combinations. (C) Spearman correlations between IA levels and migraine severity scores (VAS, VRS, and HIT-6). (D) Spearman correlation between IA and hs-CRP in PFO-M. (E) Subgroup analysis of IA stratified by sex, CI, aura status, and PFO anatomical features. Data are shown as mean ± SEM. Significance is determined by Student’s *t* test (A) or Spearman correlation (C and D).

Peripheral blood specimens from another 110 PFO-M and 110 age- and sex-matched controls were collected, and Table 4 shows the baseline data. PFO-M exhibited higher prevalence of CI, lower HGB and HDL-C, and elevated high-sensitivity C reactive protein (hs-CRP) levels compared to controls. The plasma IA levels were measured by ELISA after plasma separation, and the results suggested that PFO-M had significantly lower plasma IA levels than controls (57.01 ± 21.55 vs. 72.93 ± 28.14 ng/L, Figure 7A). In our included patients, univariate logistic regression analysis identified IA, HGB and HDL-C as protective factors, while CI and hs-CRP emerged as risk factors. After adjustment for confounders, multivariable logistic regression analysis maintained the significance of IA, HGB, and HDL-C as independent protective factors, while CI and hs-CRP remained significant risk factors for PFO-M (Table 5). Furthermore, the discriminative performance of IA, HGB, HDL-C, and hs-CRP were analyzed using ROC curves (Figure 7B). Among the four indicators, IA exhibited a moderate diagnostic value for PFO-M (AUC: 0.647, 95% CI: 0.574–0.719), with a sensitivity of 47.27% and specificity of 80.01% at cutoff 54.29 ng/L. Additionally, hs-CRP showed a moderate discriminative performance for distinguishing PFO-migraine patients from controls (AUC: 0.639, 95% CI: 0.564–0.713), with a sensitivity of 60.17% and specificity of 67.27% at cutoff 0.91 mg/L. To explore whether diagnostic discrimination could be improved, we incorporated HGB, HDL-C or hs-CRP with IA, which are potentially involved in migraine-related metabolic and inflammatory pathways,^24^ into the combined indicators. Notably, the combination of IA and hs-CRP suggested improved discriminative performance than either marker alone (AUC: 0.722, 95% CI: 0.653–0.790), with a sensitivity of 57.27% and a specificity of 82.73%. These findings suggest a potential complementary association between microbiota-derived metabolites and pro-inflammatory markers in PFO-associated migraine, indicating that the IA/hs-CRP combination may be informative for distinguishing affected patients in this cohort.

**Table 4:** Baseline demographic and clinical characteristics of PFO-M and healthy controls

Variable	Con (*n* = 110)	PFO-M (*n* = 110)	*p* value
Gender, female, n (%)	78 (70.9%)	90 (81.8%)	0.057
Age (years)	41.30 ± 12.07	39.25 ± 12.22	0.213
BMI (kg/m^2^)	22.39 ± 2.67	22.55 ± 3.54	0.712
Smokers, n (%)	15 (13.6%)	12 (10.9%)	0.538
Diabetes, n (%)	1 (0.9%)	4 (3.6%)	0.175
Hypertension, n (%)	9 (8.18%)	11 (10%)	0.815
Hyperlipidemia, n (%)	10 (9.1%)	14 (12.7%)	0.387
Cerebral infarction, n (%)	3 (2.7%)	30 (27.3%)	<0.001
White blood cells, ×109/L	5.95 ± 1.69	5.56 ± 1.48	0.069
Hemoglobin, g/L	136.69 ± 16.29	131.25 ± 16.44	0.015
Platelets, ×109/L	207.85 ± 53.08	199.39 ± 56.97	0.256
Triglyceride (mmol/L)	1.46 ± 1.59	1.71 ± 2.94	0.437
Total cholesterol (mmol/L)	4.33 ± 0.65	4.29 ± 1.03	0.787
LDL-C (mmol/L)	2.48 ± 0.57	2.43 ± 0.61	0.524
HDL-C (mmol/L)	1.40 ± 0.33	1.31 ± 0.30	0.038
Glucose (mmol/L)	4.93 ± 0.86	4.89 ± 0.96	0.763
Urea acid (μmol/L)	293.53 ± 75.75	288.39 ± 64.68	0.589
Serum creatinine (μmol/L)	64.90 ± 10.58	62.19 ± 11.72	0.257
hs-CRP (mg/L)	0.92 ± 0.75	1.39 ± 1.01	<0.001
LA (mm)	31.82 ± 3.84	30.92 ± 3.90	0.086
RA (mm)	41.35 ± 4.06	40.46 ± 4.0	0.103

Categorical data are shown as count and percentage. Quantitative data are shown as mean ± SD.

**Table 5:** Logistic regression analysis of the risk factors of PFO-M

Factor	Single factor logistic regression analysis		Multivariable logistic regression analysis	
	OR (95%CI)	*p* value	OR (95%CI)	*p* value
IA	0.97 (0.96–0.98)	<0.001	0.97 (0.96–0.98)	<0.001
Cerebral infarction	13.38 (3.94–45.38)	<0.001	12.34 (3.41–44.72)	<0.001
HGB	0.98 (0.96–0.99)	0.017	0.97 (0.95–0.99)	0.008
HDL-C	0.41 (0.17–0.96)	0.040	0.28 (0.10–0.83)	0.021
hs-CRP	1.85 (1.33–2.58)	<0.001	1.64 (1.13–2.38)	0.009

Furthermore, plasma IA levels showed significant inverse correlations with migraine severity scores (r = −0.273, −0.311 and −0.314 of VAS, VRS and HIT-6, respectively) (Figure 7C). Gut microbiota-dependent tryptophan metabolites are considered to inhibit the release of pro-inflammatory factors and inactivate the trigeminal neurovascular system.^21^ Therefore, we performed correlation analysis between plasma IA and hs-CRP in PFO-M, and a negative relationship was observed (Figure 7D), suggesting that IA-mediated anti-inflammatory effects could be involved in the PFO shunt-associated migraine pathophysiology. Subgroup stratification revealed that patients with CI or female gender exhibited significantly reduced plasma IA levels, while no statistically significant IA variations were observed between subgroups stratified by aura symptoms or PFO shunt complexity (Figure 7E). Accordingly, our findings suggest that IA may emerge as a candidate metabolic biomarker for PFO-M, warranting further investigation into its role within dysregulated tryptophan metabolism and inflammatory pathways.

## Discussion

Migraine, characterized by dysregulated neurotransmitter homeostasis, exacerbated oxidative stress and neurovascular inflammation, represents a leading cause of neurological disability worldwide. PFO is increasingly recognized as a potential pathogenic contributor, possibly through paradoxical embolism-mediated cerebral hypoperfusion or systemic circulation of vasoactive substances bypassing pulmonary filtration. While PFO closure has demonstrated therapeutic potential, the pathophysiological mechanisms remain incompletely understood. To our knowledge, this study presents an initial systematic metabolomic profiling of PFO-comorbid migraine patients, identifying systemic perturbations in mitochondrial energy metabolism, neurosteroid biosynthesis, and tryptophan catabolism that were observed in this subpopulation compared to healthy controls. Our integrated analyses indicate an association between PFO-comorbid migraine and these metabolic alterations, warranting further investigation. Arterial-venous concordance analyses further supported IA and ALC as potential biomarkers. Subgroup analyses identified sex- and aura-specific metabolic signatures, including female-driven depletion of neurosteroids and tryptophan metabolism, alongside aura-associated BCAAs dysregulation. Notably, reduced plasma levels of the gut microbiota-derived tryptophan metabolite IA were confirmed in an independent, expanded PFO-M cohort, and showed inverse associations with symptom severity, and may facilitate patient stratification. Although 5-hydroxytryptamine (5-HT) and calcitonin gene-related peptide (CGRP) remain well-established mediators in migraine pathogenesis, our exploratory data suggest that IA merits further investigation for its potential role in migraine management.

Untargeted metabolomic analysis revealed 211 DEMs. Key findings included reductions in BCAAs (e.g., ketoleucine, L-isoleucine), indole derivatives (e.g., IS, IA), sulfated neurosteroids (e.g., pregnanolone sulfate, DHEA-S), and bile acids (e.g., GCA, GCDCA), alongside elevated ketone bodies (e.g., 2-hydroxybutyric acid). Our study provides the first evidence of a marked reduction in circulating IA, a gut microbiota-derived AhR ligand, may support emerging theories of gut-brain axis interactions in alleviating neuroinflammation.^25^ Strikingly, we also should notice the downregulation of melatonin and PANA. Melatonin is a neurohormone that performs several functions, including chronobiotic, antioxidant, oncostatic, immune modulating, normothermal, and anxiolytic functions.^26^ Exogenous melatonin supplementation has been suggested to be effective for episodic migraine prophylaxis.^27^ In addition, kynurenine metabolites could promote the central sensitization of the caudal trigeminal nucleus and activate CSD, while KYNA and its analogues may serve as a potential therapeutic approach in migraine.^28^ 5-HT was not detected in our plasma analyses, which could be due to its rapid platelet uptake or enzymatic degradation. However, we observed reduction in 5-HIAA, a downstream metabolite of 5-HT in the PFO-migraine group, may reflect impaired tryptophan metabolism. Therefore, the observed depletion of IA in PFO-M may not represent an isolated metabolite-specific alteration but rather suggest the possibility of a broader disruption of microbial tryptophan-indole metabolism. This possibility warrants further investigation using targeted and integrative analyses.

Beyond IA, several additional metabolite classes associated with migraine severity warrant biological consideration. Prior studies have linked depletion of BCAAs to cortical hyperexcitability in migraine models, potentially through mitochondrial dysfunction and impaired detoxification pathways.^29^ Consistent with this framework, ALC, a key regulator of mitochondrial energy metabolism and fatty acid transport, has been reported to exert neuroprotective and anti-inflammatory effects in neurological disorders.^30^^,^^31^ The depletion of ALC, a metabolite necessary for energy metabolism and essential fatty acid anabolism, mirrors observations in chronic migraine cohorts where energy deficits exacerbate neurovascular instability.^32^ Randomized controlled trials support ALC supplementation for ameliorating neurological disorders and headache episodes.^33^^,^^34^ Similarly, decreased levels of amino acids such as alanine, valine, and isoleucine may reflect disturbances in energy substrate availability and neurotransmitter metabolism, given their roles in tricarboxylic acid cycle replenishment and central nervous system signaling, alterations that have been repeatedly reported in metabolomic studies of migraine and other neuroinflammatory conditions. In parallel, alterations in sulfated neurosteroids, including DHEA-S and dihydrotestosterone sulfate, may influence neuronal excitability and pain modulation through their known actions on GABAergic and glutamatergic signaling pathways.^35^^,^^36^ Notably, recent studies have demonstrated that PFO closure may enhance 5-HT metabolism and alleviate migraine symptoms,^37^ suggesting that tryptophan-related and neurotransmitter-associated metabolic changes may be biologically relevant to shunt-related migraine improvement. Genome-wide association studies (GWASs) of migraine have consistently implicated genes involved in neurotransmitter regulation, particularly glutamatergic signaling, ion channel function, and neurovascular pathways.^38^^,^^39^ In this context, the observed perturbations in glutamate, GABA, and catecholamine-related metabolites may be relevant, as imbalance between excitatory and inhibitory neurotransmission is thought to lower the threshold for cortical spreading depolarization (CSD) and trigeminovascular activation. Although the present study does not directly assess genetic variation, the convergence of metabolomic alterations with pathways highlighted by migraine GWAS suggests that metabolic dysregulation may represent a downstream or context-dependent manifestation of underlying genetic susceptibility in PFO-associated migraine.

These metabolic signatures are consistent with the concept that PFO may be associated with migraine through systemic metabolic alterations. We further propose a pathophysiological hypothesis in which dysregulated gut-brain signaling, altered neurosteroid milieu, and neurotransmitter imbalance play pivotal roles. In this context, the strong arterial-venous concordance, together with the correlations between arterial IA/ALC levels and migraine severity, suggests that metabolites reaching the cerebral circulation could be particularly relevant to neurological manifestations. Additionally, several metabolites exhibited relatively large fold changes between groups. However, the strong arterial-venous concordance and the consistency across LC-MS and GC-MS platforms support a biological rather than purely technical origin of these differences. Accordingly, how alterations in arterial metabolite profiles influence neural function warrants further investigation, including longitudinal assessment of metabolomic changes before and after PFO closure.

Comparative analysis identified 211 DEMs, among which 32 showed nominally significant associations with migraine symptom severity (assessed via validated clinical scoring systems). Inverse associations between ALC, IA, and migraine severity scores highlight their potential as biomarkers for treatment response monitoring. Paradoxically, residual neurosteroid levels correlated positively with headache severity, a phenomenon observed in chronic pain syndromes where compensatory upregulation of estrogen receptor and neuroinflammation occurs.^40^ Altogether, the strong arterial-venous metabolite concordance supports the use of venous sampling for CNS-related metabolic profiling, yet the paradoxical neurosteroid-pain correlations highlight the need for cerebrospinal fluid (CSF) investigations.

Considering sex-specific metabolic differences, we further examined metabolic patterns potentially relevant to hormone-sensitive migraine in PFO-M. Female patients exhibited lower circulating levels of several sulfated neurosteroids, including pregnanolone sulfate (GABAA receptor modulator), and DHEA-S, despite their higher prevalence of migraine.^41^ PFO-driven shunting may facilitate systemic circulation of vasoactive neurosteroids bypassing pulmonary metabolism, amplifying trigeminovascular excitability via GABAergic or estrogen receptorβ (Erβ)-mediated pathways.^35^^,^^42^ Females, with inherently higher shunt prevalence or volumetric flow, might exhibit accelerated depletion of pregnanolone sulfate and DHEAS due to increased cerebral endothelial uptake or compensatory catabolism. The resulting counterbalance between neuroexcitation and neuroinhibition may partially underlie the absence of markedly exacerbated migraine symptom severity observed in females.^43^^,^^44^ These findings are consistent with known physiological sexual dimorphism in androgen-related metabolites and support the technical robustness of the metabolomic measurements. At the same time, such differences may not be specific to migraine pathophysiology and could partially reflect baseline sex hormone status. Accordingly, the observed sex-associated metabolic patterns should be interpreted with caution. Larger studies incorporating sex-matched healthy controls will be required to distinguish physiological variation from disease-related metabolic alterations.

Although CSD and glutamatergic hyperexcitability are central mechanisms underlying migraine aura, PFO-mediated microemboli or shunting of vasoactive substances may further modulate oxidative stress and neurosteroid-related pathways in susceptible subgroups. In this study, aura-positive PFO-M patients exhibited distinct alterations in several plasma metabolites compared with non-aura patients. Specifically, elevated 4-hydroxyproline, an indicator of collagen turnover and oxidative stress, may reflect endothelial dysfunction or altered extracellular matrix remodeling in aura-positive PFO-M. In parallel, reduced levels of 2-hydroxybutyric acid, a marker of mitochondrial ketogenesis, suggest altered bioenergetic responses potentially relevant to neurovascular instability. These findings are consistent with prior observations linking metabolic stress to aura susceptibility.^45^ Consistent with patterns observed in sex-stratified analyses, decreased levels of N,N-diethylglycine, L-isoleucine, and ketoleucine in aura-positive PFO-M may indicate disturbances in amino acid-dependent energy metabolism and redox balance, processes that could contribute to neuronal hyperexcitability during aura episodes. Notably, reductions in ALC and IA were directionally consistent across aura and sex subgroups, suggesting a potential convergence of impaired mitochondrial bioenergetics and altered gut-brain axis-related inflammatory modulation in individuals prone to aura. Given the limited sample size, these findings should be interpreted as exploratory and hypothesis-generating. Future studies using larger cohorts and experimental models will be required to determine whether modulation of IA- or ALC-related pathways influences CSD susceptibility in a sex-dependent manner.

Given the inverse association between IA and migraine symptom severity, coupled with the methodological accessibility, we validated its significant reduction in a large-scale PFO-M cohort, exploring its potential relevance as a diagnostic biomarker. Furthermore, inverse correlations between IA and inflammatory markers were observed, consistent with a possible link between IA and inflammatory pathways in migraine. Notably, the lower IA levels were observed in patients with CI, which implying its dual role in migraine and cerebrovascular pathophysiology. These findings advocate for personalized IA supplementation strategies by dietary tryptophan intake and gut microbiota. However, these results should be interpreted as hypothesis-generating due to the observational design, and the IA-hs-CRP axis warrants validation in prospective cohorts to confirm its pathophysiological and diagnostic significance.

The observed metabolic perturbations in this study align with but extend prior work. The coordinated downregulation of BCAA catabolism, bile acids, and neurosteroids points to a unifying hypothesis: PFO-related RLS may chronically expose the brain to humoral factors (e.g., platelet microemboli, and inflammatory cytokines) that disrupt BBB integrity and mitochondrial function. Depleted ALC limits astrocytic fatty acid β-oxidation, reducing ATP supply for glutamate reuptake and promoting CSD. Notably, reduced IA levels may impair anti-inflammatory signaling and exacerbate trigeminal nociception, potentially amplified by PFO-associated gut hypoxia, consistent with emerging evidence of gut metabolite dysregulation in neuroinflammatory conditions. Sulfated neurosteroids regulate BBB permeability via organic anion transporters. Their depletion may enhance neuronal exposure to circulating metabolites, amplifying cortical hyperexcitability, resonates with reports linking neurosteroid fluctuations to menstrual migraine.^23^^,^^43^

Prior genetic studies have suggested that migraine shares a genetic basis with lipid and fatty acid metabolism. For example, specific circulating metabolites such as LPE(20:4) and DHA have shown evidence of causal involvement in migraine risk.^10^ Unfortunately, in the present study, no significant differences in these two metabolites were observed. In addition, another genetic study has reported that genes regulating HDL-C levels are significantly overrepresented among migraine risk genes, highlighting a strong shared biological basis between lipid metabolism and migraine susceptibility.^46^ This finding is consistent with our observation of reduced HDL-C levels in the larger PFO-M cohort, which remained significant after multivariable adjustment. These observations suggest that genetic variation may partially contribute to the observed phenotypic abnormalities, given that PFO, a congenital structural heart anomaly, has been reported to have a genetic basis.^47^ Further studies are needed to clarify this relationship.

In conclusion, our findings provide initial evidence suggesting mitochondrial dysfunction and tryptophan metabolism disruption as a possible mechanisms linking PFO-shunted metabolites to migraine disability. Our study provides a foundation for future work to understand the pathophysiology of the disease, advocating for the imperative for personalized therapeutic strategies in this pathophysiologically distinct migraine subtype.

### Limitations of the study

Several limitations should be acknowledged. First, this study used a two-arm design comparing patients with PFO-associated migraine and healthy controls. The lack of additional comparator groups—migraine patients without PFO and individuals with PFO but without migraine—limits our ability to determine whether the observed metabolic alterations are driven by migraine, PFO-related shunting, or their interaction. Therefore, the identified metabolic patterns should be interpreted as associations with the PFO-migraine phenotype rather than disease-specific mechanisms. In addition, the cross-sectional design precludes causal inference, and longitudinal studies or experimental PFO models are needed to clarify directionality.

Second, the untargeted discovery cohort was underpowered to detect small-effect-size metabolites across 4,858 comparisons, a common challenge in untargeted metabolomics. Although multiple metabolites were identified, independent validation was performed only for indoleacrylic acid (IA). IA was prioritized based on FDR-adjusted significance, consistent associations with migraine severity, and biological relevance to tryptophan metabolism and inflammation. Other metabolites should therefore be considered exploratory and require validation in larger, targeted studies.

Third, several potential confounding factors may have affected the study results. Dietary intake, use of acute migraine medications (e.g., NSAIDs or triptans), and timing of sampling relative to migraine attacks can influence circulating metabolite levels but were not systematically controlled. Although all samples were collected under fasting conditions, long-term diet and medication exposure could not be fully accounted for. Circulating IA levels may also be influenced by gut microbial composition, intestinal barrier function, and lifestyle factors. Future studies integrating microbiome profiling (e.g., *Lactobacillus vaginalis*) will be important to further elucidate gut-brain axis involvement.^48^

## Resource availability

### Lead contact

Further information and requests for resources and reagents should be directed to and will be fulfilled by the lead contact, Cong Lu (lucong100@126.com).

### Materials availability

This study did not generate new unique reagents.

### Data and code availability

• The raw metabolomics data generated in this study have been deposited in the MetaboLights: MTBLS13630 and are publicly available as of the date of publication.
• This paper does not report original code.
• Any additional information is available from the lead contact upon reasonable request.

## Acknowledgments

This work was supported by grants from the 10.13039/501100001809National Natural Science Foundation of China (grant no: 82470477), the Medical Association of Sichuan, China (grant no: Q22066) and the 10.13039/501100004829Science and Technology Department of Sichuan Province, China (grant no: 2023NSFSC0580).

## Author contributions

T.L. contributed to perform the data analysis and write the manuscript. Q.X. and Q.W. contributed to the clinical data collection. Y.W. and R.H. performed the metabolomic profiling and data analysis. X.W. were responsible for ELISA experiments. Y.Z. and H.H. were responsible for optimizing the figures. J.Z. and C.L. contributed to the study conception and design and revised the manuscript. All the authors read and approved the final version of the manuscript.

## Declaration of interests

The authors declare that they have no competing financial interests.

## STAR★Methods

### Key resources table

**Table:** 

REAGENT or RESOURCE	SOURCE	IDENTIFIER
**Biological samples**		

Venous and arterial blood samples	Sichuan Provincial People’s Hospital	Approval No: ChiCTR2400079772

**Chemicals, peptides, and recombinant proteins**		

Methanol	Fisher Scientific	A452-4
Acetonitrile	Fisher Scientific	A998-4
Formic acid	Fisher Scientific	A117-50
L-2-Chlorophenylalanine (internal standard)	Shanghai Hengchuang Biotechnology	C2001
Succinic acid-d4 (internal standard)	Sigma-Aldrich	293075-1G
L-Valine-d8 (internal standard)	Shanghai Haoyuan Chemexpress	HY-I1124
Cholic acid-d4 (internal standard)	Shanghai Yuanye Bio-Technology	S22155-50mg
Chloroform	Shanghai Titan Scientific	G75915B
n-Hexane	Anpel Laboratory Technologies	4.011518.0500
Pyridine	Aladdin Biochemical Technology	P141169-1L
BSTFA (with 1% TMCS)	TCI Chemicals	B0830-25ml
O-Methoxyamine hydrochloride (97%)	Shanghai Macklin Biochemical	M813479-25g
Methyl octanoate (analytical standard)	Dr. Ehrenstorfer GmbH	G162300
Methyl nonanoate (C9:0 standard)	NU-Chek Prep	N-9M-AU4-B
Methyl decanoate (C10:0 standard)	NU-Chek Prep	N-10M-A18-D
Methyl laurate (C12:0 standard)	NU-Chek Prep	N-12M-AU15-D
Methyl myristate (C14:0 standard)	NU-Chek Prep,	N-14M-A24-E
Methyl palmitate (C16:0 standard)	Dr. Ehrenstorfer GmbH	G161798
Methyl stearate (C18:0 standard)	NU-Chek Prep	N-18M-O9-C
Methyl arachidate (C20:0 standard)	NU-Chek Prep	N-20M-J27-E
Methyl behenate (C22:0 standard)	NU-Chek Prep	N-22M-JY30-E
Methyl lignocerate (C24:0 standard)	NU-Chek Prep	N-24M-S6-A

**Critical commercial assays**		

IA ELISA Kit	Enzyme Immuno	MM-928248O1

**Deposited data**		

Metabolomics	MetaboLights	MetaboLights: MTBLS13630; https://www.ebi.ac.uk/metabolights/MTBLS13630

**Software and algorithms**		

GraphPad Prism v9.3	Graphpad	N/A
SPSS v29.0	SPSS	N/A

**Other**		

Microplate reader	SpectraMax iD3	N/A
Ultrasonic cleaner	Fuyang Technology (Shenzhen)	N/A
Vortex mixer	Shanghai Hanon Instrument	N/A
High-speed refrigerated centrifuge	Shanghai Lu Xiangyi Centrifuge Instrument	N/A
Freeze centrifugal concentrator	Taicang Huamei Biochemical Instrument Factory	N/A
Air bath thermostatic shaker	Shanghai Lichen Bangxi Instrument Technology	N/A
Vacuum drying oven	Shanghai Huitai equipment Manufacturing	N/A
LC–MS system	Fisher Scientific	N/A
HPLC column	Waters Corporation	N/A
GC–MS system	Agilent Technologies	N/A
GC column	Agilent Technologies	N/A

### Experimental model and study participant details

#### Study population

This study was conducted in accordance with the Declaration of Helsinki and the study design was approved by the Ethical Committee at Sichuan Provincial People’s Hospital (Approval No.: ChiCTR2400079772). Written informed consent was obtained from all participants, and all included patients were Asians from a single center. For the untargeted metabolomic analysis via liquid or gas chromatography/tandem mass spectrometry (LC-MS/MS and GC-MS), a total of 30 migraine patients with PFO, termed PFO-M in this study, and 17 age- and sex-matched healthy individuals without PFO and migraine history were enrolled in the study between April 2023 to March 2024. For the validation experiment using enzyme-linked immunosorbent assay (ELISA), another 110 PFO-M and 110 age- and sex-matched controls were enrolled between January 2024 and March 2025. All PFO-M were evaluated by at least two experts from the Cardiovascular and Neurology Center. Inclusion criteria for PFO-M were: (i) age between 18 and 65 years; (ii) Transesophageal echocardiography (TEE) revealed an oblique separation between the septum primum and septum secundum; (iii) Contrast transthoracic echocardiography (cTTE) with agitated saline contrast under Valsalva maneuver, demonstrating right-to-left shunt (RLS) ≥30 microbubbles within three cardiac cycles; (iv) migraine was strictly categorized according to the International Classification of Headache Disorders, 3rd edition (ICHD-3)^49^; (v) ≥2 migraine attacks per month in the preceding 3 months. Inclusion criteria for control groups were: (i) age between 18 and 65 years; (ii) no history of migraine or other primary headaches; (iii) negative PFO screening by cTTE. Exclusion criteria were: (i) the presence of psychiatric co-morbidities, immunological disorders, endocrine and neurological disorders; (ii) presence of intracranial vascular malformations and space-occupying lesions; (iii) presence of carotid artery stenosis or significant intracranial arterial stenosis (>30% luminal narrowing); (iv) presence of other cardiac shunts (e.g., atrial septal defect); (v) presence of any infectious diseases; (vi) presence of heart failure, renal dysfunction or malignancy. Patients with PFO were further stratified into two subgroups based on the presence of migraine aura: (i) with aura (MA): patients reporting ≥1 typical aura symptom (e.g., visual scotoma, sensory disturbances) preceding ≥50% of migraine attacks and lasting 5–60 minutes with full reversibility (code 1.2.1); (ii) without aura (MO): patients experiencing migraine attacks without aura manifestations (code 1.1). In the study, migraine-related disability was quantified using three validated scales: (i) Visual analog scale (VAS): Patients rated pain intensity from 0 (“no pain”) to 10 (“worst imaginable pain”) during acute attacks; (ii) Verbal rating scale (VRS): Headache severity was categorized into three levels: mild (1), moderate (2), and severe (3); (iii) Headache impact test-6 (HIT-6): Functional disability was assessed using the 6-item questionnaire, with total scores ranging from 36 to 78 (higher scores indicating greater impact).

#### Blood collection

Venous and arterial blood samples were collected from PFO-M using heparin-coated vacutainer tubes, while the healthy controls provided venous blood only. All participants underwent fasting overnight (> 12 hours) prior to morning phlebotomy to minimize circadian and dietary confounding. Blood samples were centrifuged at 1000×g for 10 minutes at 4°C within 1 hour post-collection to separate cellular components, and the supernatant was further centrifuged at 16,000×g for 10 minutes at 4°C to remove residual platelets and microparticles. The final plasma supernatant was stored at -80°C until further analysis.

### Method details

#### Sample preparation for untargeted metabolomics

Plasma aliquots (150 μL) were subjected to methanol/acetonitrile (2:1, v/v) extraction with internal standards (acetyl-d3-carnitine, phenylalanine-3,3-d2, tiapride, trazodone, reserpine, phytosphingosine, and chlorpromazine) at 4:1 solvent-to-sample ratio. After vortexing (10 min, 4°C) and overnight protein precipitation (-40°C), supernatants were centrifuged (12,000×g, 10 min, 4°C) and filtered through 0.22 μm organic phase syringe filters. For GC-MS analysis, dried extracts (nitrogen evaporator) underwent two-step derivatization: (i) Methoximation: 80 μL methoxyamine hydrochloride (15 mg/mL in pyridine) at 37°C for 60 min; (ii) Silylation: 50 μL BSTFA + 20 μL n-hexane + 10 μL fatty acid internal standard mixture (C8-C24 in chloroform) at 70°C for 60 min. Quality control (QC) samples were generated by pooling equal volumes of all extracts.

#### Chromatographic conditions and mass spectrometry acquisition

LC-MS/MS: System: Waters ACQUITY UPLC I-Class Plus coupled to Thermo Q Exactive HF; Column: HSS T3 (100×2.1 mm, 1.8 μm); Mobile phase: 0.1% formic acid (A)/acetonitrile (B); Gradient: 2% B (0-1 min) → 98% B (1-10 min) → 2% B (12.1-15 min); Flow rate: 0.35 mL/min; Injection: 3 μL; Full MS (m/z 70-1050, 15,000 resolution) with top-10 MS/MS (stepped NCE 10/20/40 eV).

GC-MS: System: Agilent 7890B-5977A; Column: DB-5MS (30 m×0.25 mm×0.25 μm); Program: 60°C (0.5 min) → 270°C (8°C/min) → 305°C (20°C/min, 5 min); EI source (70 eV); Scan range: m/z 50-500.

#### Metabolomics data processing

LC-MS: Raw files processed in Progenesis QI v3.0 (Nonlinear Dynamics) for: Baseline correction; Peak picking (5 ppm mass error); Retention time alignment (max shift 0.5 min). Filtering criteria: Remove features with >50% missing values per group; Score ≥36 (0–80 scale) for compound annotation.

GC-MS: Converted to .abf format via AnalysisBaseFileConverter; Processed in MS-DIAL v4.8 with: Deconvolution (minimum peak height: 5000 amplitude); Library matching (NIST 20 & in-house database), normalization (internal standard RSD <10%); Filtering criteria: Score ≥70 (0–100 scale); Remove derivatization artifacts. Data Integration: Merged LC-MS (positive/negative ion modes) and GC-MS datasets into a unified matrix using in-house Python scripts.

#### Identify significantly altered metabolic features

Principal Component Analysis (PCA) assessed analytical stability and global metabolic variation. Orthogonal Partial Least Squares-Discriminant Analysis (OPLS-DA) was utilized to distinguish the overall differences in metabolic profiles between groups and to identify differential metabolites among the groups. A combination of multivariate and univariate analysis is employed to screen for differential metabolites between groups. In OPLS-DA analyses, the Variable Importance in Projection (VIP) value is used to measure the impact and explanatory power of each metabolite's expression pattern on the classification and discrimination of samples between groups. Subsequently, a t-test is utilized to verify the significance of the differences between groups (p-value < 0.05 and VIP > 1). Pathway enrichment analysis utilized KEGG (https://www.genome.jp/kegg/) and Reactome (https://reactome.org/) databases.

#### Measurement of the IA level

Plasma IA levels were measured using a competitive ELISA kit (Enzyme Immuno Co., Ltd, Jiangsu, China). Briefly, plasma samples from 110 PFO-M and 110 healthy controls were thawed on ice, centrifuged (10,000×g, 10 min, 4°C) and analyzed per manufacturer protocol. In this assay, free IA competes with HRP-conjugated IA for antibody binding on pre-coated plates. After washing, tetramethylbenzidine substrate was added, and reactions were stopped prior to measuring absorbance at 450 nm.

### Quantification and statistical analysis

Quantitative data in tables are presented as mean ± SD and categorical data are expressed as percentages. Quantitative data in figures are presented as mean ± SEM. Continuous variables were analyzed using independent Student's t-tests for normally distributed data or Mann-Whitney U tests for non-parametric comparisons between two groups. For the untargeted metabolomic analysis (4,858 metabolites), a false discovery rate (FDR) adjustment was further applied, with q < 0.1 considered significant. Categorical variables were assessed through χ^2^ tests. Bivariate correlations used Spearman’s ρ coefficients (without multiple-testing correction). Univariate logistic regression analysis was performed to identify potential protective or risk factors, and ROC curve analysis was utilized to evaluate the potential diagnostic performance for discriminating PFO-M from controls. All analyses were conducted using SPSS v29.0, with two-tailed p-value < 0.05 considered statistically significant.

#### Statistical analysis for untargeted metabolomics and false positive control

Given the exploratory nature and high dimensionality of untargeted metabolomic data (4,858 features), we employed a conservative, multi-tiered strategy to ensure robust identification of differential metabolites and control the false discovery rate. Differential metabolites were initially identified using combined criteria of a Variable Importance in Projection (VIP) score > 1.0 from Orthogonal Partial Least Squares-Discriminant Analysis (OPLS-DA) and a univariate p-value < 0.05 (Student’s t-test). To address multiple testing, a false discovery rate (FDR) adjustment was applied to the p-values of these candidate metabolites using the Benjamini-Hochberg procedure, with an FDR-corrected q-value < 0.10 set as the significance threshold for defining differentially expressed metabolites (DEMs). All subsequent pathway enrichment analyses and the selection of key metabolites for downstream investigation (e.g., IA, ALC) were based on this FDR-corrected list. Furthermore, the risk of overfitting in the OPLS-DA model was assessed through permutation testing (200 iterations), which confirmed model validity (negative Q^2^ intercept). Finally, to provide the strongest safeguard against spurious findings, our primary candidate metabolite, indoleacrylic acid (IA), was subjected to targeted validation in a large, independent cohort (n=220) using ELISA.

## Supplemental information

Table S1. Association Between Levels of 211 DEMs and Headache Scores in PFO-M

## References

[1] Ashina M., Katsarava Z., Do T.P., Buse D.C., Pozo-Rosich P., Özge A., Krymchantowski A.V., Lebedeva E.R., Ravishankar K., Yu S. (2021). Migraine: epidemiology and systems of care. Lancet.

[2] Liu K., Wang B.Z., Hao Y., Song S., Pan M. (2020). The Correlation Between Migraine and Patent Foramen Ovale. Front. Neurol..

[3] Shi F., Sha L., Li H., Tang Y., Huang L., Liu H., Li X., Li L., Yang W., Kang D., Chen L. (2023). Recent progress in patent foramen ovale and related neurological diseases: A narrative review. Front. Neurol..

[4] Tobis J.M., Charles A., Silberstein S.D., Sorensen S., Maini B., Horwitz P.A., Gurley J.C. (2017). Percutaneous Closure of Patent Foramen Ovale in Patients With Migraine: The PREMIUM Trial. J. Am. Coll. Cardiol..

[5] Wilmshurst P.T., Nightingale S., Walsh K.P., Morrison W.L. (2000). Effect on migraine of closure of cardiac right-to-left shunts to prevent recurrence of decompression illness or stroke or for haemodynamic reasons. Lancet.

[6] Reisman M., Christofferson R.D., Jesurum J., Olsen J.V., Spencer M.P., Krabill K.A., Diehl L., Aurora S., Gray W.A. (2005). Migraine headache relief after transcatheter closure of patent foramen ovale. J. Am. Coll. Cardiol..

[7] Ben-Assa E., Rengifo-Moreno P., Al-Bawardy R., Kolte D., Cigarroa R., Cruz-Gonzalez I., Sakhuja R., Elmariah S., Pomerantsev E., Vaina L.M. (2020). Effect of Residual Interatrial Shunt on Migraine Burden After Transcatheter Closure of Patent Foramen Ovale. JACC Cardiovasc. Interv..

[8] Mojadidi M.K., Kumar P., Mahmoud A.N., Elgendy I.Y., Shapiro H., West B., Charles A.C., Mattle H.P., Sorensen S., Meier B. (2021). Pooled Analysis of PFO Occluder Device Trials in Patients With PFO and Migraine. J. Am. Coll. Cardiol..

[9] Zhao E.F., Xie H., Zhang Y.S. (2023). Identification of potential influencing factors associated with elimination of migraine headache in patients with PFO after percutaneous closure. Zhonghua Xinxueguanbing Zazhi.

[10] Tanha H.M., Sathyanarayanan A., Nyholt D.R., International Headache Genetics Consortium (2021). Genetic overlap and causality between blood metabolites and migraine. Am. J. Hum. Genet..

[11] Körtési T., Spekker E., Vécsei L. (2022). Exploring the Tryptophan Metabolic Pathways in Migraine-Related Mechanisms. Cells.

[12] Liu J., Xi K., Zhang L., Han M., Wang Q., Liu X. (2024). Tryptophan metabolites and gut microbiota play an important role in pediatric migraine diagnosis. Headache Pain.

[13] Domitrz I., Koter M.D., Cholojczyk M., Domitrz W., Baranczyk-Kuzma A., Kaminska A. (2015). Changes in Serum Amino Acids in Migraine Patients without and with Aura and their Possible Usefulness in the Study of Migraine Pathogenesis. CNS Neurol. Disord.: Drug Targets.

[14] Lynch C.J., Adams S.H. (2014). Branched-chain amino acids in metabolic signalling and insulin resistance. Nat. Rev. Endocrinol..

[15] Zorumski C.F., Paul S.M., Izumi Y., Covey D.F., Mennerick S. (2013). Neurosteroids, stress and depression: potential therapeutic opportunities. Neurosci. Biobehav. Rev..

[16] Wlodarska M., Luo C., Kolde R., d'Hennezel E., Annand J.W., Heim C.E., Krastel P., Schmitt E.K., Omar A.S., Creasey E.A. (2017). Indoleacrylic Acid Produced by Commensal Peptostreptococcus Species Suppresses Inflammation. Cell Host Microbe.

[17] Fila M., Chojnacki J., Derwich M., Chojnacki C., Pawlowska E., Blasiak J. (2024). Urine 5-Hydroxyindoleacetic Acid Negatively Correlates with Migraine Occurrence and Characteristics in the Interictal Phase of Episodic Migraine. Int. J. Mol. Sci..

[18] Charles A.C., Baca S.M. (2013). Cortical spreading depression and migraine. Nat. Rev. Neurol..

[19] Qi Z., Zhou L., Dai S., Zhang P., Zhong H., Zhou W., Zhao X., Xu H., Zhao G., Wu H., Ge J. (2025). Intermittent fasting inhibits platelet activation and thrombosis through the intestinal metabolite indole-3-propionate. Life Metab..

[20] Sarzi-Puttini P., Giorgi V., Di Lascio S., Fornasari D. (2021). Acetyl-L-carnitine in chronic pain: A narrative review. Pharmacol. Res..

[21] Papetti L., Del Chierico F., Frattale I., Toto F., Scanu M., Mortera S.L., Rapisarda F., Di Michele M., Monte G., Ursitti F. (2024). Pediatric migraine is characterized by traits of ecological and metabolic dysbiosis and inflammation. J. Headache Pain.

[22] Allais G., Chiarle G., Sinigaglia S., Airola G., Schiapparelli P., Benedetto C. (2020). Gender-related differences in migraine. Neurol. Sci..

[23] Nappi R.E., Tiranini L., Sacco S., De Matteis E., De Icco R., Tassorelli C. (2022). Role of Estrogens in Menstrual Migraine. Cells.

[24] van Welie F.C., Kreft L.A., Huisman J.M.A., Terwindt G.M. (2023). Sex-specific metabolic profiling to explain the increased CVD risk in women with migraine: a narrative review. J. Headache Pain.

[25] Sun J., Zhang Y., Kong Y., Ye T., Yu Q., Kumaran Satyanarayanan S., Su K.P., Liu J. (2022). Microbiota-derived metabolite Indoles induced aryl hydrocarbon receptor activation and inhibited neuroinflammation in APP/PS1 mice. Brain Behav. Immun..

[26] Zduńska A., Cegielska J., Domitrz I. (2022). The Pathogenetic Role of Melatonin in Migraine and Its Theoretic Implications for Pharmacotherapy: A Brief Overview of the Research. Nutrients.

[27] Tseng P.T., Yang C.P., Su K.P., Chen T.Y., Wu Y.C., Tu Y.K., Lin P.Y., Stubbs B., Carvalho A.F., Matsuoka Y.J. (2020). The association between melatonin and episodic migraine: A pilot network meta-analysis of randomized controlled trials to compare the prophylactic effects with exogenous melatonin supplementation and pharmacotherapy. J. Pineal Res..

[28] Török N., Majláth Z., Fülöp F., Toldi J., Vécsei L. (2016). Brain Aging and Disorders of the Central Nervous System: Kynurenines and Drug Metabolism. Curr. Drug Metab..

[29] Ren C., Liu J., Zhou J., Liang H., Wang Y., Sun Y., Ma B., Yin Y. (2018). Low levels of serum serotonin and amino acids identified in migraine patients. Biochem. Biophys. Res. Commun..

[30] Guerreiro G., Deon M., Becker G.S., Dos Reis B.G., Wajner M., Vargas C.R. (2025). Neuroprotective effects of L-carnitine towards oxidative stress and inflammatory processes: a review of its importance as a therapeutic drug in some disorders. Metab. Brain Dis..

[31] da Silva L.E., de Oliveira M.P., da Silva M.R., Abel J.D.S., Tartari G., de Aguiar da Costa M., Ludvig Gonçalves C., Rezin G.T. (2023). L-carnitine and Acetyl-L Carnitine: A Possibility for Treating Alterations Induced by Obesity in the Central Nervous System. Neurochem. Res..

[32] Kilinc Y.B., Kilinc E., Danis A., Hanci F., Turay S., Ozge A., Bolay H. (2023). Mitochondrial metabolism related markers GDF-15, FGF-21, and HIF-1α are elevated in pediatric migraine attacks. Headache.

[33] Hershman D.L., Unger J.M., Crew K.D., Minasian L.M., Awad D., Moinpour C.M., Hansen L., Lew D.L., Greenlee H., Fehrenbacher L. (2013). Randomized double-blind placebo-controlled trial of acetyl-L-carnitine for the prevention of taxane-induced neuropathy in women undergoing adjuvant breast cancer therapy. J. Clin. Oncol..

[34] Chiechio S., Copani A., Nicoletti F., Gereau R.W. (2006). L-acetylcarnitine: a proposed therapeutic agent for painful peripheral neuropathies. Curr. Neuropharmacol..

[35] Krivoshein G., Rivera-Mancilla E., MaassenVanDenBrink A., Giniatullin R., van den Maagdenberg A.M.J.M. (2025). Sex difference in TRPM3 channel functioning in nociceptive and vascular systems: an emerging target for migraine therapy in females?. J. Headache Pain.

[36] Vitku J., Hill M., Kolatorova L., Kubala Havrdova E., Kancheva R. (2022). Steroid Sulfation in Neurodegenerative Diseases. Front. Mol. Biosci..

[37] Dong B., Li X., Zhang L., Liang G., Zheng W., Gui L., Ji S., Tang Y., Li H., Li W. (2025). Effects of patent foramen ovale in migraine: a metabolomics-based study. J. Physiol..

[38] Grangeon L., Lange K.S., Waliszewska-Prosół M., Onan D., Marschollek K., Wiels W., Mikulenka P., Farham F., Gollion C., Ducros A., European Headache Federation School of Advanced Studies EHF-SAS (2023). Genetics of migraine: where are we now?. J. Headache Pain.

[39] Dias A., Mariz T., Sousa A., Lemos C., Alves-Ferreira M. (2022). A review of migraine genetics: gathering genomic and transcriptomic factors. Hum. Genet..

[40] Xu Z., Xie W., Feng Y., Wang Y., Li X., Liu J., Xiong Y., He Y., Chen L., Liu G., Wu Q. (2022). Positive interaction between GPER and β-alanine in the dorsal root ganglion uncovers potential mechanisms: mediating continuous neuronal sensitization and neuroinflammation responses in neuropathic pain. J. Neuroinflammation.

[41] Sacco S., Ricci S., Degan D., Carolei A. (2012). Migraine in women: the role of hormones and their impact on vascular diseases. J. Headache Pain.

[42] Dourson A.J., Darken R.S., Baranski T.J., Gereau R.W., Ross W.T., Nahman-Averbuch H. (2024). The role of androgens in migraine pathophysiology. Neurobiol. Pain.

[43] Rustichelli C., Bellei E., Bergamini S., Monari E., Lo Castro F., Baraldi C., Tomasi A., Ferrari A. (2021). Comparison of pregnenolone sulfate, pregnanolone and estradiol levels between patients with menstrually-related migraine and controls: an exploratory study. J. Headache Pain.

[44] Rustichelli C., Monari E., Avallone R., Bellei E., Bergamini S., Tomasi A., Ferrari A. (2021). Dehydroepiandrosterone sulfate, dehydroepiandrosterone, 5α-dihydroprogesterone and pregnenolone in women with migraine: Analysis of serum levels and correlation with age, migraine years and frequency. J. Pharm. Biomed. Anal..

[45] Zielman R., Postma R., Verhoeven A., Bakels F., van Oosterhout W.P.J., Meissner A., van den Maagdenberg A.M.J.M., Terwindt G.M., Mayboroda O.A., Ferrari M.D. (2016). Metabolomic changes in CSF of migraine patients measured with 1H-NMR spectroscopy. Mol. Biosyst..

[46] Tanha H.M., Martin N.G., Whitfield J.B., Nyholt D.R., International Headache Genetics Consortium IHGC (2021). Association and genetic overlap between clinical chemistry tests and migraine. Cephalalgia.

[47] Dong B., Li Y., Ai F., Geng J., Tang T., Peng W., Tang Y., Wang H., Tian Z., Bu F., Chen L. (2024). Genetic variation in patent foramen ovale: a case-control genome-wide association study. Front. Genet..

[48] Wang Z., Liu T., Liu L., Xie J., Tang F., Pi Y., Zhong Y., He Z., Zhang W., Zheng C. (2025). Lactobacillus vaginalis alleviates DSS induced colitis by regulating the gut microbiota and increasing the production of 3-indoleacrylic acid. Pharmacol. Res..

[49] (2018). Headache Classification Committee of the International Headache Society (IHS) The International Classification of Headache Disorders, 3rd edition. Cephalalgia.

